# Association between triglyceride-glucose index and its combination with obesity indicators and depression: findings from NHANES 2005–2020

**DOI:** 10.3389/fpsyt.2025.1533819

**Published:** 2025-03-10

**Authors:** Hongli Sun, Wei He, Jingyu Bu, Huifang Zhang, Huimei Huang, Kai Ma

**Affiliations:** ^1^ Shaanxi Institute for Pediatric Diseases, Xi’an Key Laboratory of Children’s Health and Diseases, Xi’an Children’s Hospital (Affiliated Children’s Hospital of Xi’an Jiaotong University), Xi’an, Shaanxi, China; ^2^ Department of Laboratory, Xi’an Children’s Hospital (Affiliated Children’s Hospital of Xi’an Jiaotong University), Xi’an, Shaanxi, China; ^3^ Department of Pediatrics, Second Affiliated Hospital, Air Force Medical University, Xi’an, Shaanxi, China; ^4^ Department of Emergency, Xi’an Children’s Hospital (Affiliated Children’s Hospital of Xi’an Jiaotong University), Xi’an, Shaanxi, China; ^5^ Department of Nephrology, Xi’an Children’s Hospital (Affiliated Children’s Hospital of Xi’an Jiaotong University), Xi’an, Shaanxi, China

**Keywords:** depression, triglyceride-glucose (TyG) index, triglyceride-glucose-waist circumference (TyG-WC), triglyceride-glucose-waist height ratio (TyG-WHtR), triglyceride-glucose-body mass index (TyG-BMI)

## Abstract

**Background:**

The relationship between the triglyceride-glucose (TyG) index, its combination with obesity indicators, and depression remains understudied in the American population.

**Methods:**

This cross-sectional study analyzed data from 10,423 adults in the National Health and Nutrition Examination Survey (NHANES) conducted between 2005 and 2020. We employed multivariable logistic regression analysis, smoothing techniques, generalized additive models, stratified analyses, and sensitivity analyses to examine the relationship between TyG, its combination (TyG-WC, TyG-WHtR, TyG-BMI) with obesity indicators, and depression.

**Results:**

The results indicate that the TyG index, TyG-WC, TyG-WHtR, TyG-BMI, and depression exhibited a significant statistical association with depressive symptoms (all P for trend < 0.001). Specifically, a one-unit increase in the TyG index correlated with a 37% increase in the risk of depressive symptoms (95% CI: 1.21–1.55), a one-unit increase in TyG-WC correlated with a 3.26 times increase in the risk of depressive symptoms (95% CI: 2.22–4.80), a one-unit increase in TyG-WHtR correlated with a 27% increase in the risk of depressive symptoms (95% CI: 1.18–1.36), and a one-unit increase in TyG-BMI correlated with a 2.30 times increase in the risk of depressive symptoms (95% CI: 1.72–3.08). There was a significant nonlinear correlation between TyG-WC, TyG-WHtR, and TyG-BMI with depressive symptoms (all P for nonlinearity < 0.001), except for a linear correlation between the TyG index and depressive symptoms (P for linearity < 0.001).

**Conclusion:**

Monitoring the TyG index, TyG-WC, TyG-WHtR, TyG-BMI may facilitate depression risk assessment and prevention.

## 1 Introduction

Depression, affecting approximately 280 million people worldwide, represents a significant global health concern according to the World Health Organization (WHO) (1). This condition not only impairs daily functioning but also has profound consequences for individuals and society (2). Depression is closely associated with chronic health problems such as cardiovascular diseases (3), obesity (4), and diabetes (5), and is a leading risk factor for suicide (6). The economic burden of depression is substantial, contributing to lost productivity, increased absenteeism, and higher healthcare expenditures (7). Effective intervention and early detection are crucial for managing depression’s long-term consequences (8). Identifying predictive factors for depression risk can help target high-risk individuals, emphasizing the importance of early disease prevention through mental health screenings and proactive support.

Recent studies have demonstrated an association between the triglyceride glucose (TyG) index and depression (9–13). The TyG index, calculated as Ln (fasting triglycerides [TG, mg/dL] × fasting blood glucose [FBG, mg/dL]/2), is a measure of insulin resistance. Insulin resistance, characterized by reduced cellular sensitivity to insulin, can lead to type 2 diabetes (14), obesity (15) and depression (16). Some studies have confirmed a U-shaped relationship between TyG index and depression, suggesting that both low and high levels of TyG index may increase depressive symptoms risk (9, 17, 18). However, recent research indicates that TyG index combined with obesity indices may be more effective in assessing depression than the TyG index alone (10).

Obesity rates are rising globally, posing significant health risks including cardiovascular diseases (19), type 2 diabetes (20), and mental health disorders (21). TyG index combined with obesity indices is closely associated with depression (10, 22, 23). Previous reports have focused on body mass index (BMI), waist-to-height ratio (WHtR), and waist circumference. The calculation formulas for TyG index combined with obesity indices are as follows (1): TyG-BMI = TyG × body mass (kg)/height² (m²) (2); TyG-WHtR = TyG × waist circumference (cm)/height (cm) (3); TyG-WC = TyG × waist circumference (cm) (24).

Despite some studies reporting correlations between TyG index combined with obesity indices and depression, these studies have not adequately addressed population selection and the dose-dependent relationship between these indices and depression (10, 11, 17). Therefore, our study aims to expand the sample size to accurately investigate these relationships and explore their dose-dependent nature.

## 2 Materials and methods

### 2.1 Data source and study population

**Figure 1:**
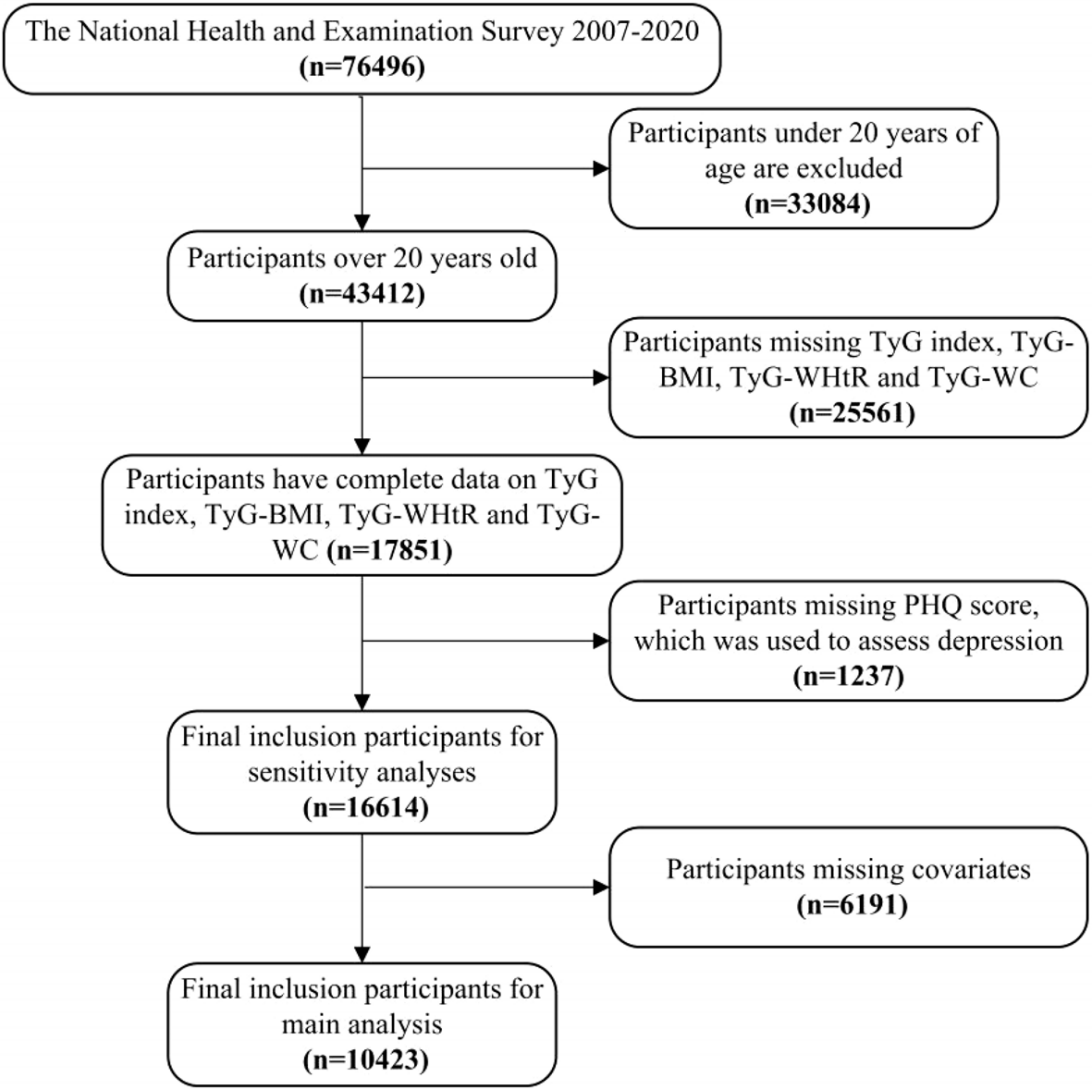
Flowchart depicting the participants’ selection. TyG, Triglyceride-glucose index; TyG-WC, Triglyceride-glucose-waist circumference; TyG-WHtR, Triglyceride-glucose-waist height ratio; TyG-BMI, Triglyceride-glucose-body mass index.

This study utilized data from the National Health and Nutrition Examination Survey (NHANES) conducted between 2005 and 2020. NHANES employs a complex, multistage, probabilistic sampling design to ensure national representativeness. The initial study population included participants with complete data on TyG index, obesity indicators, and depression measures. Exclusion criteria included missing data on TyG index, TyG-WC, TyG-WHtR, TyG-BMI, depression, and covariates. The final analytical cohort consisted of 10,423 individuals. Flowchart depicting the participants’ selection was shown in **Figure 1**. The Ethics Review Committee of the National Center for Health Statistics (NCHS) approved the NHANES research plan, and all participants provided written informed consent.

### 2.2 Definitions of TyG index, TyG−WC, TyG−WHtR, and TyG−BMI

Fasting blood glucose (FBG) and triglyceride levels were assessed through blood samples. Body weight, height, and waist measurements were recorded during physical examinations. BMI and waist-to-height ratio were calculated from these measurements. The exposure variables were calculated as follows (1): TyG = ln[triglycerides (mg/dl) × glucose (mg/dl)/2] (25) (2); BMI = body mass (kg)/height² (m²) (3); WHtR = waist circumference/height (4); TyG-WC = TyG × waist circumference; TyG-WHtR = TyG × WHtR; TyG-BMI = TyG × BMI (24). Participants were divided into quartiles (Q1, Q2, Q3, Q4) based on these indices, with Q1 as the reference group.

#### 2.2.1 Identification of depression

Depressive symptoms were identified using the Patient Health Questionnaire-9 (PHQ-9), administered two weeks prior to examination. The PHQ-9 is a reliable self-report screening tool assessing depressive symptoms severity based on DSM criteria. It has been extensively validated, demonstrating a sensitivity and specificity of 88% (26).It consists of 9 items scored from 0 (not at all) to 3 (nearly every day), with a total score ranging from 0 to 27. Scores ≥ 10 indicate depressive symptoms (27).

#### 2.2.2 Assessment of covariates

The following covariates that may influence the association between TyG index, TyG-WC, TyG-WHtR, and TyG-BMI and depression were included. The characteristics included gender, age, race (including Mexican American, other Hispanic, non-Hispanic White, non-Hispanic Black, non-Hispanic Asian, and other races), education level (<9 years, 9–11 years, ≥12 years), marital status (married/cohabiting, widowed/divorced/separated, never married), and the ratio of family income to poverty (PIR), and waist circumference, BMI, smoking (yes/no), alcohol drinking (mild, moderate and heavy), and diabetes status (yes/no).

BMI was categorized on the basis of the WHO’s standard as < 25, 25-29.9, and ≥ 30 kg/m^2^, corresponding to a healthy weight, excess weight, and obesity, respectively (28). Smoking was defined as having smoked at least 100 cigarettes in a lifetime (29). Alcohol drinking was divided into three categories: For alcohol drinking group, current heavy alcohol use (≥3 drinks per day for females, ≥4 drinks per day for males, or binge drinking [≥4 drinks on same occasion for females, ≥5 drinks on same occasion for males] on 5 or more days per month); current moderate alcohol use (≥2 drinks per day for females, ≥3 drinks per day for males, or binge drinking ≥2 days per month); all other cases are mild (29). A fasting blood glucose (FBG) level of ≥ 125 mg/dL was considered indicators of diabetes (30).

### 2.3 Statistical analysis

Continuous variables conforming to a normal distribution were expressed as mean ± standard deviation (SD), or as median (range). Categorical variables were expressed as percentages. Baseline characteristics were described according to the quartiles of the TyG index, TyG-WC, TyG-WHtR, and TyG-BMI, respectively. Kruskal-Wallis H test were used to compare continuous variables and Fisher exact tests were used to compare categorical variables. Multivariable logistic regression analysis was performed to assess TyG index, TyG-WC, TyG-WHtR, TyG-BMI and depression. To standardize the odds ratios (OR) corresponding to the TyG-WC and TyG-BMI, we performed a Ln-transformation on the TyG-WC and TyG-BMI. Results presented as OR with 95% confidence intervals (95% CI). Initially, an unadjusted model 1 was analyzed, after which a crude adjusted model 2 was developed to account for variables including survey year, age, gender, race. Subsequently, a refined adjusted model 3 was created that incorporated additional factors, including education level, family PIR, marital status, diabetes, smoke, alcohol drinking. Smoothing techniques and generalized additive models were performed to account for any potential nonlinear relationship between TyG index, TyG-WC, TyG-WHtR, TyG-BMI and depression. In addition, stratified analyses were conducted to assess potential moderating effects of age (20-39/40-59/≥60), gender (male/female), education level (<9/9-11/≥12), family PIR (≤1.3/1.3), marital status (Married/Living with partner, Widowed/divorced/separated, Never married), smoke (yes/no), alcohol drinking (mild/moderate/heavy), and diabetes (yes/no). Interactions between the TyG index, TyG-WC, TyG-WHtR, TyG-BMI and each of the above variables were tested. After excluding missing values for independent variables and dependent variable, we used multiple imputation, based on 5 replications and a chained equation approach method in the R MI procedure, to account for missing data on education, marital status, family PIR, smoke and alcohol drinking.

All statistical analyses were performed using R (http://www.r-project.org, The R Foundation) and EmpowerStats (http://www.empowwerstats.com, X&Y Solutions, Inc., Boston, MA) software. A 2-tailed *P* < 0.05 was considered statistically significant.

## 3 Results

### 3.1 Basic characteristics of participants

Basic demographic characteristics are shown in **Table 1**. The analysis included 10,423 participants (53.45% male, 46.55% female), aged 20-85 years (mean 46.84 ± 16.95). Significant differences were observed between non-depressive symptoms and depressive symptoms groups for gender, race, education level, family PIR, marital status, BMI, WC, smoking, alcohol drinking, diabetes, PHQ score, TyG index, TyG-WC, TyG-WHtR, and TyG-BMI (all *P* < 0.05). Survey year showed no significant difference.

**Table 1:** Baseline characteristics of American adults aged 20–85 years stratified by depression and non-depression, NHANES 2005–2020.

	Total (n = 10423)	Non-depression (n = 9592)	Depression (n = 831)	P-value
Survey year				0.107
2005-2006	1219 (11.70%)	1148 (11.97%)	71 (8.54%)	
2007-2008	1415 (13.58%)	1297 (13.52%)	118 (14.20%)	
2009-2010	1525 (14.63%)	1393 (14.52%)	132 (15.88%)	
2011-2012	1339 (12.85%)	1239 (12.92%)	100 (12.03%)	
2013-2014	1456 (13.97%)	1337 (13.94%)	119 (14.32%)	
2015-2016	1244 (11.94%)	1143 (11.92%)	101 (12.15%)	
2017-2020	2225 (21.35%)	2035 (21.22%)	190 (22.86%)	
Gender				<0.001
Male	5571 (53.45%)	5242 (54.65%)	329 (39.59%)	
Female	4852 (46.55%)	4350 (45.35%)	502 (60.41%)	
**Age (year)**	46.84 ± 16.95	46.88 ± 17.11	46.39 ± 15.08	0.426
Race				<0.001
Mexican American	1499 (14.38%)	1391 (14.50%)	108 (13.00%)	
Other Hispanic	945 (9.07%)	845 (8.81%)	100 (12.03%)	
Non-Hispanic White	4845 (46.48%)	4482 (46.73%)	363 (43.68%)	
Non-Hispanic Black	2093 (20.08%)	1897 (19.78%)	196 (23.59%)	
Other Race	1041 (9.99%)	977 (10.19%)	64 (7.70%)	
Education level (year)				<0.001
< 9	649 (6.23%)	571 (5.95%)	78 (9.39%)	
09-Nov	1262 (12.11%)	1120 (11.68%)	142 (17.09%)	
≥12	8512 (81.67%)	7901 (82.37%)	611 (73.53%)	
**Family PIR**	2.74 ± 1.65	2.82 ± 1.64	1.81 ± 1.46	<0.001
Marital status				<0.001
Married/Living with partner	6365 (61.07%)	6006 (62.61%)	359 (43.20%)	
Widowed/divorced/separated	2027 (19.45%)	1746 (18.20%)	281 (33.81%)	
Never married	2031 (19.49%)	1840 (19.18%)	191 (22.98%)	
**BMI (kg/m^2^)**	28.99 ± 6.79	28.84 ± 6.66	30.67 ± 7.97	<0.001
**WC (cm)**	99.06 ± 16.46	98.77 ± 16.31	102.45 ± 17.78	<0.001
Smoke				<0.001
No	5272 (50.58%)	4983 (51.95%)	289 (34.78%)	
Yes	5151 (49.42%)	4609 (48.05%)	542 (65.22%)	
Alcohol drinking				<0.001
Mild	5140 (49.31%)	4819 (50.24%)	321 (38.63%)	
Moderate	2309 (22.15%)	2125 (22.15%)	184 (22.14%)	
Heavy	2974 (28.53%)	2648 (27.61%)	326 (39.23%)	
Diabetes				<0.001
No	9268 (88.92%)	8565 (89.29%)	703 (84.60%)	
Yes	1155 (11.08%)	1027 (10.71%)	128 (15.40%)	
**PHQ**	3.07 ± 4.11	2.12 ± 2.38	14.04 ± 3.86	<0.001
**TyG index**	8.58 ± 0.67	8.57 ± 0.67	8.72 ± 0.72	<0.001
**TyG-WC**	854.20 ± 176.91	850.40 ± 174.70	897.98 ± 195.47	<0.001
**TyG-WHtR**	5.06 ± 1.04	5.03 ± 1.02	5.39 ± 1.19	<0.001
**TyG-BMI**	250.03 ± 66.51	248.38 ± 65.07	268.99 ± 78.93	<0.001

### 3.2 Relationship between TyG index, TyG−WC, TyG−WHtR, TyG−BMI and depression

The association of TyG index, TyG−WC, TyG−WHtR, TyG−BMI with depressive symptoms are shown in **Table 2**. The association between the TyG index, TyG−WC, TyG−WHtR, TyG−BMI and depressive symptoms was examined through multiple regression analyses, considering the TyG index TyG−WC, TyG−WHtR, TyG−BMI as both a continuous and categorical variable (divided into quartiles, with the first quartile as the reference). In the non-adjusted model, adjust I model and adjust II model, the TyG index, TyG−WC, TyG−WHtR, TyG−BMI and depressive symptoms exhibited a significant statistical association with depressive symptoms (all *P* for trend < 0.001). Specifically, a one-unit increase in the TyG index correlated with a 37% increase in the risk of depressive symptoms (95% CI: 1.21-1.55), a one-unit increase in the TyG-WC correlated with a 3.26 times increase in the risk of depressive symptoms (95% CI: 2.22-4.80), a one-unit increase in the TyG−WHtR correlated with a 27% increase in the risk of depressive symptoms (95% CI: 1.18-1.36), and a one-unit increase in the TyG−BMI correlated with a 2.30 times increase in the risk of depressive symptoms (95% CI: 1.72-3.08). Furthermore, when compared to participants in the lowest TyG index quartile, those in the highest quartile had a 72% increase in the risk of depressive symptoms (95% CI: 1.37-2.17). When compared to participants in the lowest TyG-WC quartile, those in the highest quartile had a 82% increase in the risk of depressive symptoms (95% CI: 1.46-2.27). When compared to participants in the lowest TyG-WHtR quartile, those in the highest quartile had a 72% increase in the risk of depressive symptoms (95% CI: 1.37-2.15). When compared to participants in the lowest TyG−BMI quartile, those in the highest quartile had a 60% increase in the risk of depressive symptoms (95% CI: 1.29-1.97).

**Table 2:** Multivariate-adjusted association of TyG and its combination with obesity indicators and depression in US adults 18–85 years, NHANES 2005–2020.

	Non-adjusted	Adjust I	Adjust II
**TyG index**	1.35 (1.22, 1.50) <0.0001	1.57 (1.42, 1.75) <0.0001	1.37 (1.21, 1.55) <0.0001
TyG quartile			
Q1	1.0	1.0	1.0
Q2	1.06 (0.86, 1.32) 0.5856	1.22 (0.98, 1.52) 0.0769	1.10 (0.88, 1.38) 0.4142
Q3	1.14 (0.92, 1.40) 0.2390	1.41 (1.13, 1.75) 0.0026	1.21 (0.96, 1.52) 0.1064
Q4	1.69 (1.39, 2.06) <0.0001	2.27 (1.83, 2.81) <0.0001	1.72 (1.37, 2.17) <0.0001
**TyG-WC**	3.40 (2.40, 4.81) <0.0001	4.84 (3.38, 6.92) <0.0001	3.26 (2.22, 4.80) <0.0001
TyG-WC quartile			
Q1	1.0	1.0	1.0
Q2	1.05 (0.84, 1.30) 0.6915	1.16 (0.93, 1.46) 0.1860	1.09 (0.87, 1.37) 0.4620
Q3	1.22 (0.98, 1.50) 0.0736	1.45 (1.16, 1.81) 0.0011	1.31 (1.05, 1.65) 0.0187
Q4	1.86 (1.53, 2.27) <0.0001	2.25 (1.82, 2.77) <0.0001	1.82 (1.46, 2.27) <0.0001
**TyG-WHtR**	1.37 (1.28, 1.46) <0.0001	1.38 (1.29, 1.47) <0.0001	1.27 (1.18, 1.36) <0.0001
TyG-WHtR quartile			
Q1	1.0	1.0	1.0
Q2	0.90 (0.71, 1.13) 0.3507	0.96 (0.76, 1.22) 0.7482	0.95 (0.75, 1.21) 0.6764
Q3	1.33 (1.08, 1.65) 0.0076	1.48 (1.18, 1.84) 0.0006	1.40 (1.12, 1.75) 0.0037
Q4	2.01 (1.65, 2.45) <0.0001	2.13 (1.73, 2.63) <0.0001	1.72 (1.37, 2.15) <0.0001
**TyG-BMI**	2.91 (2.21, 3.82) <0.0001	2.86 (2.17, 3.76) <0.0001	2.30 (1.72, 3.08) <0.0001
TyG-BMI quartile			
Q1	1.0	1.0	1.0
Q2	0.89 (0.72, 1.11) 0.3133	0.96 (0.77, 1.20) 0.7125	0.98 (0.78, 1.23) 0.8306
Q3	1.09 (0.88, 1.35) 0.4192	1.17 (0.94, 1.45) 0.1598	1.13 (0.90, 1.41) 0.2902
Q4	1.77 (1.46, 2.15) <0.0001	1.81 (1.48, 2.21) <0.0001	1.60 (1.29, 1.97) <0.0001

β (95%CI) P value/OR (95%CI) P value.

Non-adjusted model adjust for: None.

Adjust I model adjust for: Survey year; Age; Gender; Race.

Adjust II model adjust for: Survey year; Age; Gender; Race; Education level; Family PIR; Marital status; Smoke; Alcohol drinking; Diabetes.

### 3.3 Nonlinear relationship between TyG index, TyG−WC, TyG−WHtR, TyG−BMI and depression

**Figure 2:**
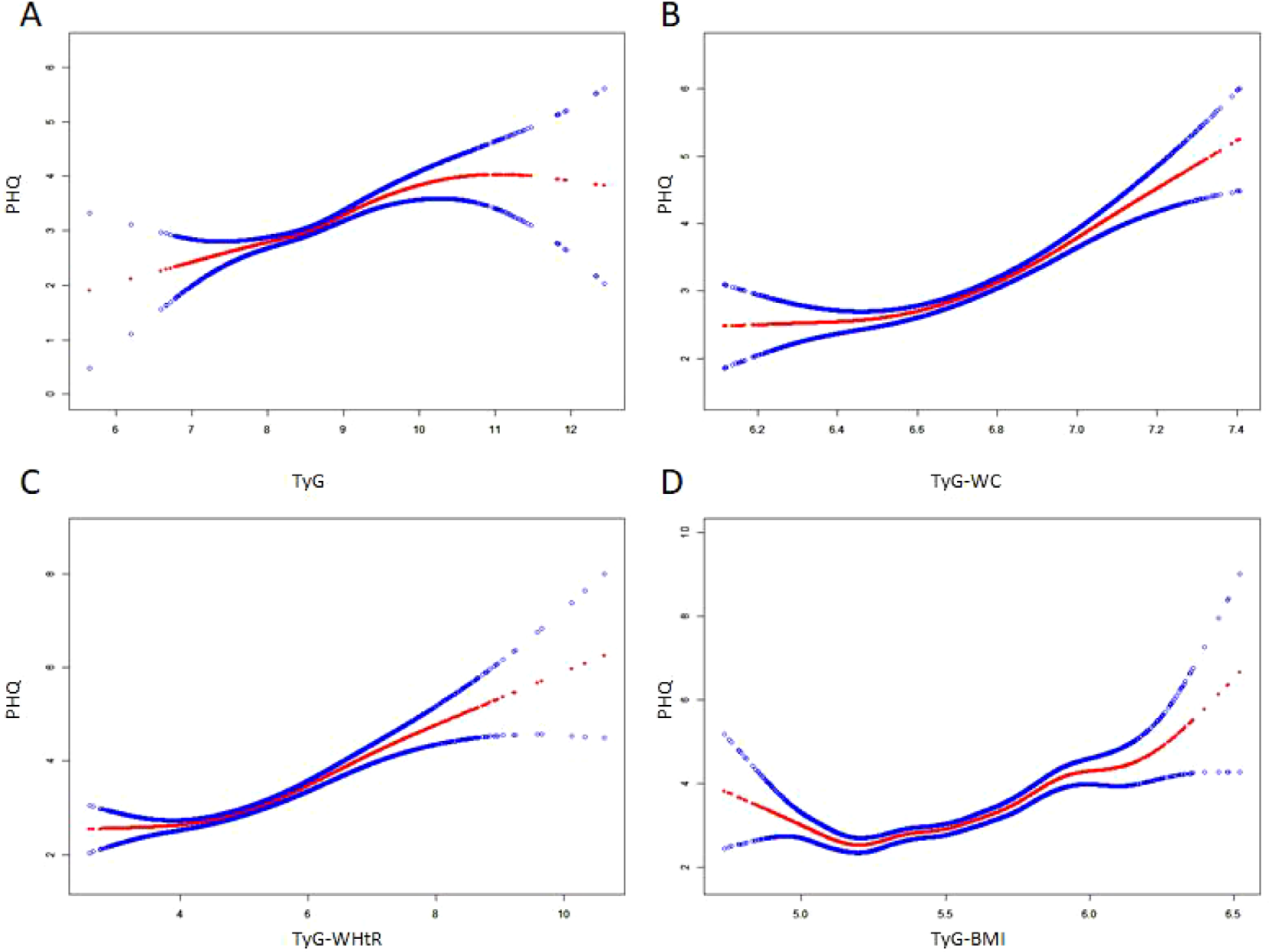
The non-linear relationship between TyG index and its combination with obesity indicators and depressive symptoms. A non-linear relationship between them was identified after adjusting for survey year; age; gender; race; education level; family pir; marital status; smoke; alcohol drinking; diabetes. The solid red line represents the smooth curve fit between variables. Blue bands represent the 95% of confidence interval from the fit. **(A)** TyG, Triglyceride-glucose index; **(B)** TyG-WC, Triglyceride-glucose-waist circumference; **(C)** TyG-WHtR, Triglyceride-glucose-waist height ratio; **(D)** TyG-BMI, Triglyceride-glucose-body mass index.

A smooth curve fitting approach and threshold effect analysis were used to detect the potentially non-linear relationship between TyG index, TyG−WC, TyG−WHtR, TyG−BMI with depressive symptoms (**Figure 2**; **Table 3**). There was a significant nonlinear correlation between TyG-WC, TyG−WHtR, TyG−BMI with depressive symptoms (all P for nonlinearity < 0.001) except for a linear correlation between TyG and depressive symptoms (P for linearity < 0.001). The results of threshold effect analysis were shown in **Table 3**. The two-piecewise regression model showed the inflection point of TyG index, TyG-WC, TyG−WHtR, TyG−BMI was 8.51, 6.66, 4.14, and 5.47 respectively, after fully adjusted for covariates (survey year, age, gender, race, education level, family PIR, marital status, diabetes, smoke, alcohol drinking). On the left side of the inflection point, the effect size had no statistical significance on TyG index (OR, 1.25; 95% CI, 0.95–1.64; P = 0.1062), TyG-WC (OR, 1.02; 95% CI, 0.39-2.66; P = 0.9691), TyG−WHtR (OR, 0.79; 95% CI, 0.52-1.19; P = 0.2600), TyG−BMI (OR, 0.81; 95% CI, 0.43-1.54; P = 0.5227). On the right side of the inflection point, the risk of depressive symptoms increased 44% in one-unit of TyG index (OR, 1.44; 95% CI, 1.21-1.71; P < 0.0001), increased 5.73 times in one-unit of TyG-WC (OR, 5.37; 95% CI, 3.22-10.21; P < 0.0001), increased 33% in one-unit of TyG−WHtR (OR, 1.33; 95% CI, 1.22-1.45; P = 0.0233), increased 4.47 times in one-unit of TyG−BMI (OR, 4.47; 95% CI,2.80-7.15; P = 0.0005).

**Table 3:** Threshold analysis for the association of TyG and its combination with obesity indicators and depression in US adults 18–85 years, NHANES 2005–2020.

	TyG index	TyG-WC	TyG-WHtR	TyG-BMI
Model I				
**One line slope**	1.37 (1.21, 1.55) <0.0001	3.26 (2.22, 4.80) <0.0001	1.27 (1.18, 1.36) <0.0001	2.30 (1.72, 3.08) <0.0001
Model II				
**Turning point (K)**	8.51	6.66	4.14	5.47
< K	1.25 (0.95, 1.64) 0.1062	1.02 (0.39, 2.66) 0.9691	0.79 (0.52, 1.19) 0.2600	0.81 (0.43, 1.54) 0.5227
≥ K	1.44 (1.21, 1.71) <0.0001	5.73 (3.22, 10.21) <0.0001	1.33 (1.22, 1.45) <0.0001	4.47 (2.80, 7.15) <0.0001
β between Segment 1 and Segment 2	1.15 (0.80, 1.66) 0.4510	5.62 (1.49, 21.19) 0.0107	1.68 (1.07, 2.64) 0.0233	5.51 (2.12, 14.36) 0.0005
**Logarithmic likelihood ratio test**	0.453	0.012	0.027	<0.001

OR (95%CI) P value.

Adjust for: Survey year; Age; Gender; Race; Education level; Family PIR; Marital status; Smoke; Alcohol drinking; Diabetes.

### 3.4 Stratification of TyG index, TyG−WC, TyG−WHtR, and TyG−BMI in relation to depression

**Figure 3:**
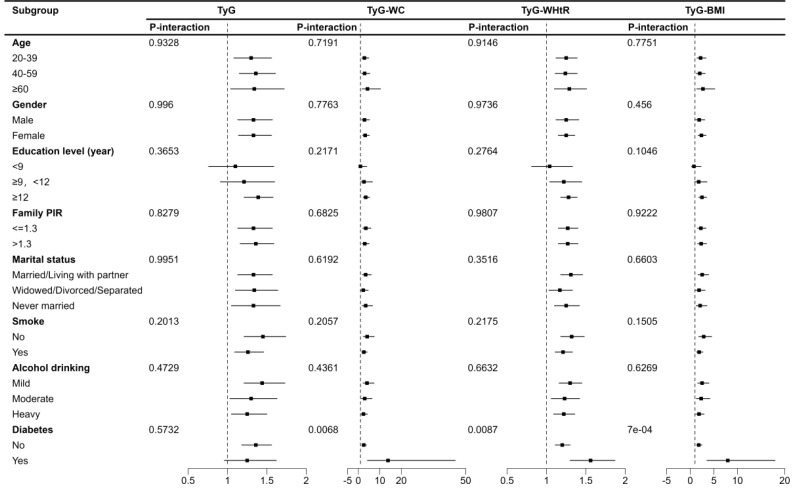
Subgroup analysis of TyG index and its combination with obesity indicators and depression in US adults 18–85 years, NHANES 2005–2020. TyG, Triglyceride-glucose index; TyG-WC, Triglyceride-glucose-waist circumference; TyG-WHtR, Triglyceride-glucose-waist height ratio; TyG-BMI, Triglyceride-glucose-body mass index.

Subgroup and interaction analysis showed significant associations between TyG index, TyG-WC, TyG-WHtR, TyG-BMI, and depression across various subgroups. No significant interactions were observed except for diabetes (**Figure 3**).

### 3.5 Sensitivity analyses

The study used multiple interpolations to impute the education, marital status, family PIR, smoke and alcohol drinking values for sensitivity analysis. **Supplementary Table 1** showed the distribution of missing variables. The direction of the results of the sensitivity analyses (**Supplementary Tables 2**, **3**) was stable consistent with the formal results.

## 4 Discussion

### 4.1 Association between TyG index and combined obesity indices with depression

This study aimed to explore the association between the triglyceride glucose (TyG) index, combined obesity indices (TyG-WC, TyG-WHtR, TyG-BMI), and depression among a national representative sample of individuals from the NHANES dataset. Our findings indicate a significant association between the TyG index and its combined obesity indices with depressive symptoms. Specifically, we observed a dose-dependent relationship in which higher TyG, TyG-WC, TyG-WHtR, and TyG-BMI levels were correlated with an increased risk of depressive symptoms. These findings offer critical insights into the potential role of insulin resistance and obesity in the etiology of depression, underscoring the relevance of metabolic health in mental well-being.

### 4.2 Mechanisms linking metabolic health and depression

Our results align with previous research indicating a positive relationship between TyG index and depression risk (31, 32). This risk could be explained by the complex relationship between metabolic disturbances and neuropsychological health. Insulin promotes glutamate neurotransmitter release by boosting the phosphorylation and membrane localization of NMDA receptor subunits (33, 34), while also promoting the surface expression of GluR1-containing AMPA receptors (35). The glutamate system plays a key role in depression, with dysregulation leading to neurotoxicity and reduced synaptic plasticity, which contribute to depressive symptoms (36–38). Additionally, high TyG values might reflect underlying metabolic syndrome (39) or inflammatory processes (40), which are known risk factors for depression (41, 42). Recent studies using advanced techniques such as high-plex protein and whole transcriptome co-mapping at cellular resolution with spatial CITE-seq have further elucidated the complex interplay between metabolic dysregulation and neuroinflammation, providing additional mechanistic insights into the link between metabolic health and depression (43, 44).

### 4.3 Combined TyG index-obesity indices as predictors of depression

Notably, we found that combining the TyG index with obesity indicators (WC, WHtR, BMI) enhances the predictive accuracy for depression risk. This finding suggests that assessing metabolic and obesity-related markers jointly could be more effective in identifying individuals at high risk for depression than using TyG index alone. Obesity is well-documented to contribute to depression through mechanisms like chronic low-grade inflammation, oxidative stress, and altered hypothalamic-pituitary-adrenal (HPA) axis function (4, 45), which could amplify the depressive effects of metabolic dysregulation. Thus, our study highlights the potential of the TyG index-obesity combined indices as novel screening tools for depression in populations with metabolic and weight-related risk factors. The nonlinear relationship between TyG index, TyG-WC, TyG-WHtR, TyG-BMI and depression revealed threshold effects. While TyG index showed a linear correlation, TyG-WC, TyG-WHtR, and TyG-BMI demonstrated nonlinear associations. Threshold analysis identified inflection points: TyG index (8.51), TyG-WC (6.66), TyG-WHtR (4.14), and TyG-BMI (5.47). Below these points, no significant association with depression was found. However, beyond the thresholds, the risk of depression increased substantially: TyG index (44% increase), TyG-WC (5.73-fold increase), TyG-WHtR (33% increase), and TyG-BMI (4.47-fold increase). These findings suggest that metabolic health, particularly waist circumference and BMI, plays a crucial role in depression risk. Early monitoring and intervention for metabolic dysfunction could help prevent depression.

### 4.4 Strengths and limitations

Our study has several strengths, including a large sample size and a robust statistical analysis that controlled for potential confounders. However, there are limitations to consider. First, the cross-sectional design precludes causative inference, as we cannot determine whether metabolic disturbances lead to depression or vice versa. Second, while we accounted for multiple confounders, other factors, such as dietary habits, physical activity, and medication use, which might influence both metabolic and mental health, were not included. Finally, the data are based on a U.S. population, limiting the generalizability of findings to other populations with different sociodemographic or genetic profiles. Additionally, we acknowledge that the PHQ-9 is a validated screening instrument rather than a clinical diagnostic tool for depression. Due to the nature of NHANES data, formal clinical diagnoses of depression were not available, which limited our ability to use clinician-assessed diagnostic criteria. We have clarified this point in the Methods and Discussion sections and explicitly stated that our study assesses “depressive symptoms” rather than “clinical depression” to avoid potential misinterpretation. We also recognize that diseases such as hypothyroidism and Parkinson’s disease may influence depressive symptoms. While NHANES does not provide comprehensive data on all potential comorbid conditions, we have now explicitly mentioned this limitation in the Discussion section and acknowledged the potential impact of undiagnosed or unadjusted comorbid conditions on our findings.

### 4.5 Conclusion

In conclusion, this study adds to the growing body of literature linking metabolic health and depressive symptoms, suggesting that TyG index combined with obesity indices may serve as a valuable predictor of depressive symptoms. Our findings support integrating metabolic and obesity assessments into mental health screenings, particularly for high-risk groups. Future longitudinal studies should investigate the causal relationship between TyG index, obesity indices, and depressive symptoms, and explore the impact of lifestyle interventions targeting metabolic health on mental health outcomes. Additionally, incorporating longitudinal data that captures various economic cycles can provide deeper insights into how different economic conditions affect mental health over time. We believe that this interdisciplinary approach will not only enhance the robustness of our findings but also offer more actionable insights for policymakers and healthcare providers. Furthermore, future research may benefit from the use of advanced statistical models that account for the dynamic interplay between economic variables and mental health outcomes. By doing so, we can better isolate the specific economic factors contributing to depression and develop targeted interventions to mitigate their impact. This holistic approach will ensure that our research remains relevant and impactful in addressing the mental health challenges posed by fluctuations in economic conditions.

## Data Availability

The original contributions presented in the study are included in the article/**Supplementary Material**. Further inquiries can be directed to the corresponding author.
