# Supplementary material for: Association between triglyceride-glucose index and its combination with obesity indicators and depression: findings from NHANES 2005–2020
Source: Front Psychiatry. 2025 Mar 10;16:1533819. doi: 10.3389/fpsyt.2025.1533819 (PMC11931011; doi:10.3389/fpsyt.2025.1533819)
Supplement: Supplementary file 1 [file Table1.docx]

Supplemental Table 1 Distribution of missing variables.

| Characteristic | Missing number |
| --- | --- |
| Education level | 11 |
| Family PIR | 1456 |
| Marital status | 6 |
| Smoke | 14 |
| Alcohol drinking | 5281 |

Supplemental Table 2 Five sets of regression data after interpolation for missing data on education, marital status, family PIR, smoke and alcohol drinking.

| Exposure | Non-adjusted | Non-adjusted | Non-adjusted | Non-adjusted | Non-adjusted | Adjust I | Adjust I | Adjust I | Adjust I | Adjust I | Adjust II | Adjust II | Adjust II | Adjust II | Adjust II |
| --- | --- | --- | --- | --- | --- | --- | --- | --- | --- | --- | --- | --- | --- | --- | --- |
| TyG | 1.34 (1.24, 1.45) <0.0001 | 1.34 (1.24, 1.45) <0.0001 | 1.34 (1.24, 1.45) <0.0001 | 1.34 (1.24, 1.45) <0.0001 | 1.34 (1.24, 1.45) <0.0001 | 1.50 (1.39, 1.63) <0.0001 | 1.50 (1.39, 1.63) <0.0001 | 1.50 (1.39, 1.63) <0.0001 | 1.50 (1.39, 1.63) <0.0001 | 1.50 (1.39, 1.63) <0.0001 | 1.30 (1.19, 1.43) <0.0001 | 1.30 (1.18, 1.43) <0.0001 | 1.30 (1.18, 1.43) <0.0001 | 1.30 (1.18, 1.43) <0.0001 | 1.30 (1.18, 1.43) <0.0001 |
| TyG quartile |  |  |  |  |  |  |  |  |  |  |  |  |  |  |  |
| Q1 | 1.0 | 1.0 | 1.0 | 1.0 | 1.0 | 1.0 | 1.0 | 1.0 | 1.0 | 1.0 | 1.0 | 1.0 | 1.0 | 1.0 | 1.0 |
| Q2 | 1.04 (0.88, 1.22) 0.6589 | 1.04 (0.88, 1.22) 0.6589 | 1.04 (0.88, 1.22) 0.6589 | 1.04 (0.88, 1.22) 0.6589 | 1.04 (0.88, 1.22) 0.6589 | 1.15 (0.97, 1.36) 0.0998 | 1.15 (0.97, 1.36) 0.0998 | 1.15 (0.97, 1.36) 0.0998 | 1.15 (0.97, 1.36) 0.0998 | 1.15 (0.97, 1.36) 0.0998 | 1.06 (0.89, 1.26) 0.4945 | 1.06 (0.89, 1.26) 0.5002 | 1.06 (0.89, 1.26) 0.5018 | 1.06 (0.89, 1.26) 0.5044 | 1.06 (0.89, 1.26) 0.4997 |
| Q3 | 1.18 (1.01, 1.39) 0.0403 | 1.18 (1.01, 1.39) 0.0403 | 1.18 (1.01, 1.39) 0.0403 | 1.18 (1.01, 1.39) 0.0403 | 1.18 (1.01, 1.39) 0.0403 | 1.39 (1.17, 1.64) 0.0002 | 1.39 (1.17, 1.64) 0.0002 | 1.39 (1.17, 1.64) 0.0002 | 1.39 (1.17, 1.64) 0.0002 | 1.39 (1.17, 1.64) 0.0002 | 1.20 (1.01, 1.42) 0.0425 | 1.19 (1.00, 1.42) 0.0449 | 1.19 (1.00, 1.42) 0.0446 | 1.19 (1.00, 1.42) 0.0440 | 1.20 (1.00, 1.42) 0.0438 |
| Q4 | 1.65 (1.42, 1.92) <0.0001 | 1.65 (1.42, 1.92) <0.0001 | 1.65 (1.42, 1.92) <0.0001 | 1.65 (1.42, 1.92) <0.0001 | 1.65 (1.42, 1.92) <0.0001 | 2.05 (1.74, 2.41) <0.0001 | 2.05 (1.74, 2.41) <0.0001 | 2.05 (1.74, 2.41) <0.0001 | 2.05 (1.74, 2.41) <0.0001 | 2.05 (1.74, 2.41) <0.0001 | 1.57 (1.32, 1.88) <0.0001 | 1.57 (1.31, 1.88) <0.0001 | 1.57 (1.31, 1.88) <0.0001 | 1.57 (1.31, 1.88) <0.0001 | 1.57 (1.31, 1.88) <0.0001 |
| TyG-WC | 3.47 (2.65, 4.54) <0.0001 | 3.47 (2.65, 4.54) <0.0001 | 3.47 (2.65, 4.54) <0.0001 | 3.47 (2.65, 4.54) <0.0001 | 3.47 (2.65, 4.54) <0.0001 | 4.41 (3.34, 5.82) <0.0001 | 4.41 (3.34, 5.82) <0.0001 | 4.41 (3.34, 5.82) <0.0001 | 4.41 (3.34, 5.82) <0.0001 | 4.41 (3.34, 5.82) <0.0001 | 3.02 (2.24, 4.06) <0.0001 | 3.01 (2.24, 4.05) <0.0001 | 3.02 (2.24, 4.06) <0.0001 | 3.00 (2.23, 4.05) <0.0001 | 3.01 (2.24, 4.06) <0.0001 |
| TyG-WC quartile |  |  |  |  |  |  |  |  |  |  |  |  |  |  |  |
| Q1 | 1.0 | 1.0 | 1.0 | 1.0 | 1.0 | 1.0 | 1.0 | 1.0 | 1.0 | 1.0 | 1.0 | 1.0 | 1.0 | 1.0 | 1.0 |
| Q2 | 1.03 (0.87, 1.22) 0.7649 | 1.03 (0.87, 1.22) 0.7649 | 1.03 (0.87, 1.22) 0.7649 | 1.03 (0.87, 1.22) 0.7649 | 1.03 (0.87, 1.22) 0.7649 | 1.10 (0.92, 1.31) 0.2816 | 1.10 (0.92, 1.31) 0.2816 | 1.10 (0.92, 1.31) 0.2816 | 1.10 (0.92, 1.31) 0.2816 | 1.10 (0.92, 1.31) 0.2816 | 1.05 (0.88, 1.25) 0.5931 | 1.05 (0.88, 1.25) 0.6007 | 1.05 (0.88, 1.25) 0.5964 | 1.05 (0.88, 1.25) 0.6006 | 1.05 (0.88, 1.25) 0.5972 |
| Q3 | 1.30 (1.10, 1.52) 0.0017 | 1.30 (1.10, 1.52) 0.0017 | 1.30 (1.10, 1.52) 0.0017 | 1.30 (1.10, 1.52) 0.0017 | 1.30 (1.10, 1.52) 0.0017 | 1.46 (1.23, 1.73) <0.0001 | 1.46 (1.23, 1.73) <0.0001 | 1.46 (1.23, 1.73) <0.0001 | 1.46 (1.23, 1.73) <0.0001 | 1.46 (1.23, 1.73) <0.0001 | 1.34 (1.13, 1.59) 0.0009 | 1.34 (1.13, 1.59) 0.0009 | 1.34 (1.13, 1.59) 0.0009 | 1.34 (1.13, 1.59) 0.0009 | 1.34 (1.13, 1.59) 0.0009 |
| Q4 | 1.82 (1.56, 2.12) <0.0001 | 1.82 (1.56, 2.12) <0.0001 | 1.82 (1.56, 2.12) <0.0001 | 1.82 (1.56, 2.12) <0.0001 | 1.82 (1.56, 2.12) <0.0001 | 2.05 (1.75, 2.41) <0.0001 | 2.05 (1.75, 2.41) <0.0001 | 2.05 (1.75, 2.41) <0.0001 | 2.05 (1.75, 2.41) <0.0001 | 2.05 (1.75, 2.41) <0.0001 | 1.68 (1.42, 1.99) <0.0001 | 1.68 (1.42, 1.99) <0.0001 | 1.68 (1.42, 1.99) <0.0001 | 1.68 (1.41, 1.99) <0.0001 | 1.68 (1.42, 1.99) <0.0001 |
| TyG-WHtR | 1.36 (1.30, 1.43) <0.0001 | 1.36 (1.30, 1.43) <0.0001 | 1.36 (1.30, 1.43) <0.0001 | 1.36 (1.30, 1.43) <0.0001 | 1.36 (1.30, 1.43) <0.0001 | 1.35 (1.28, 1.42) <0.0001 | 1.35 (1.28, 1.42) <0.0001 | 1.35 (1.28, 1.42) <0.0001 | 1.35 (1.28, 1.42) <0.0001 | 1.35 (1.28, 1.42) <0.0001 | 1.24 (1.17, 1.31) <0.0001 | 1.24 (1.17, 1.31) <0.0001 | 1.24 (1.17, 1.31) <0.0001 | 1.24 (1.17, 1.31) <0.0001 | 1.24 (1.17, 1.31) <0.0001 |
| TyG-WHtR quartile |  |  |  |  |  |  |  |  |  |  |  |  |  |  |  |
| Q1 | 1.0 | 1.0 | 1.0 | 1.0 | 1.0 | 1.0 | 1.0 | 1.0 | 1.0 | 1.0 | 1.0 | 1.0 | 1.0 | 1.0 | 1.0 |
| Q2 | 1.06 (0.90, 1.26) 0.4843 | 1.06 (0.90, 1.26) 0.4843 | 1.06 (0.90, 1.26) 0.4843 | 1.06 (0.90, 1.26) 0.4843 | 1.06 (0.90, 1.26) 0.4843 | 1.12 (0.94, 1.33) 0.2133 | 1.12 (0.94, 1.33) 0.2133 | 1.12 (0.94, 1.33) 0.2133 | 1.12 (0.94, 1.33) 0.2133 | 1.12 (0.94, 1.33) 0.2133 | 1.12 (0.94, 1.34) 0.2186 | 1.12 (0.93, 1.34) 0.2240 | 1.12 (0.93, 1.34) 0.2238 | 1.12 (0.93, 1.34) 0.2236 | 1.12 (0.93, 1.34) 0.2224 |
| Q3 | 1.25 (1.05, 1.47) 0.0099 | 1.25 (1.05, 1.47) 0.0099 | 1.25 (1.05, 1.47) 0.0099 | 1.25 (1.05, 1.47) 0.0099 | 1.25 (1.05, 1.47) 0.0099 | 1.30 (1.09, 1.55) 0.0030 | 1.30 (1.09, 1.55) 0.0030 | 1.30 (1.09, 1.55) 0.0030 | 1.30 (1.09, 1.55) 0.0030 | 1.30 (1.09, 1.55) 0.0030 | 1.22 (1.02, 1.46) 0.0269 | 1.22 (1.02, 1.46) 0.0279 | 1.22 (1.02, 1.46) 0.0275 | 1.22 (1.02, 1.46) 0.0283 | 1.22 (1.02, 1.46) 0.0275 |
| Q4 | 2.16 (1.86, 2.52) <0.0001 | 2.16 (1.86, 2.52) <0.0001 | 2.16 (1.86, 2.52) <0.0001 | 2.16 (1.86, 2.52) <0.0001 | 2.16 (1.86, 2.52) <0.0001 | 2.15 (1.83, 2.52) <0.0001 | 2.15 (1.83, 2.52) <0.0001 | 2.15 (1.83, 2.52) <0.0001 | 2.15 (1.83, 2.52) <0.0001 | 2.15 (1.83, 2.52) <0.0001 | 1.75 (1.47, 2.08) <0.0001 | 1.75 (1.47, 2.07) <0.0001 | 1.75 (1.47, 2.08) <0.0001 | 1.75 (1.47, 2.07) <0.0001 | 1.75 (1.47, 2.08) <0.0001 |
| TyG-BMI | 2.84 (2.30, 3.50) <0.0001 | 2.84 (2.30, 3.50) <0.0001 | 2.84 (2.30, 3.50) <0.0001 | 2.84 (2.30, 3.50) <0.0001 | 2.84 (2.30, 3.50) <0.0001 | 2.62 (2.13, 3.24) <0.0001 | 2.62 (2.13, 3.24) <0.0001 | 2.62 (2.13, 3.24) <0.0001 | 2.62 (2.13, 3.24) <0.0001 | 2.62 (2.13, 3.24) <0.0001 | 2.12 (1.69, 2.64) <0.0001 | 2.11 (1.69, 2.64) <0.0001 | 2.12 (1.70, 2.65) <0.0001 | 2.11 (1.69, 2.64) <0.0001 | 2.12 (1.69, 2.64) <0.0001 |
| TyG-BMI quartile |  |  |  |  |  |  |  |  |  |  |  |  |  |  |  |
| Q1 | 1.0 | 1.0 | 1.0 | 1.0 | 1.0 | 1.0 | 1.0 | 1.0 | 1.0 | 1.0 | 1.0 | 1.0 | 1.0 | 1.0 | 1.0 |
| Q2 | 0.84 (0.71, 1.00) 0.0447 | 0.84 (0.71, 1.00) 0.0447 | 0.84 (0.71, 1.00) 0.0447 | 0.84 (0.71, 1.00) 0.0447 | 0.84 (0.71, 1.00) 0.0447 | 0.87 (0.73, 1.04) 0.1327 | 0.87 (0.73, 1.04) 0.1327 | 0.87 (0.73, 1.04) 0.1327 | 0.87 (0.73, 1.04) 0.1327 | 0.87 (0.73, 1.04) 0.1327 | 0.87 (0.73, 1.04) 0.1156 | 0.87 (0.73, 1.04) 0.1148 | 0.87 (0.73, 1.04) 0.1173 | 0.87 (0.72, 1.03) 0.1126 | 0.87 (0.73, 1.04) 0.1148 |
| Q3 | 1.17 (1.00, 1.37) 0.0514 | 1.17 (1.00, 1.37) 0.0514 | 1.17 (1.00, 1.37) 0.0514 | 1.17 (1.00, 1.37) 0.0514 | 1.17 (1.00, 1.37) 0.0514 | 1.20 (1.02, 1.41) 0.0318 | 1.20 (1.02, 1.41) 0.0318 | 1.20 (1.02, 1.41) 0.0318 | 1.20 (1.02, 1.41) 0.0318 | 1.20 (1.02, 1.41) 0.0318 | 1.17 (0.99, 1.38) 0.0674 | 1.17 (0.99, 1.38) 0.0701 | 1.17 (0.99, 1.38) 0.0681 | 1.17 (0.99, 1.38) 0.0702 | 1.17 (0.99, 1.38) 0.0686 |
| Q4 | 1.78 (1.53, 2.06) <0.0001 | 1.78 (1.53, 2.06) <0.0001 | 1.78 (1.53, 2.06) <0.0001 | 1.78 (1.53, 2.06) <0.0001 | 1.78 (1.53, 2.06) <0.0001 | 1.73 (1.48, 2.01) <0.0001 | 1.73 (1.48, 2.01) <0.0001 | 1.73 (1.48, 2.01) <0.0001 | 1.73 (1.48, 2.01) <0.0001 | 1.73 (1.48, 2.01) <0.0001 | 1.52 (1.29, 1.78) <0.0001 | 1.51 (1.29, 1.78) <0.0001 | 1.52 (1.29, 1.78) <0.0001 | 1.51 (1.29, 1.78) <0.0001 | 1.52 (1.29, 1.78) <0.0001 |

Note: OR (95%CI) Pvalue
Non-adjusted model adjust for: None
Adjust I model adjust for: Age; Gender; Race; Survey Year
Adjust II model adjust for: Age; Gender; Race; Family PIR; Survey Year; Education Level; Marital Status; Smoke; Alcohol Drinking; Diabetes

Supplementary Table 3 Association of TyG and its combination with obesity indicators and depression after imputing the baseline missing values

| Exposure | Non-adjusted | Adjust I | Adjust II |
| --- | --- | --- | --- |
| TyG | 1.34 (1.24, 1.45)<0.0000 | 1.50 (1.39, 1.62)<0.0000 | 1.30 (1.18, 1.43)<0.0000 |
| TyG quartile |  |  |  |
| Q1 | 1 | 1 | 1 |
| Q2 | 1.04 (0.88, 1.22)0.6379 | 1.15 (0.97, 1.36)0.105 | 1.06 (0.89, 1.26)0.5112 |
| Q3 | 1.18 (1.01, 1.38)0.0422 | 1.39 (1.17, 1.65)0.0001 | 1.19 (1.00, 1.42)0.0465 |
| Q4 | 1.65 (1.42, 1.92)<0.0000 | 2.05 (1.74, 2.41)<0.0000 | 1.57 (1.31, 1.88)<0.0000 |
| TyG-WC | 3.47 (2.65, 4.54)<0.0000 | 4.41 (3.34, 5.82)<0.0000 | 3.01 (2.24, 4.06)<0.0000 |
| TyG-WC quartile |  |  |  |
| Q1 | 1 | 1 | 1 |
| Q2 | 1.03 (0.87, 1.22)0.7318 | 1.10 (0.92, 1.31)0.2904 | 1.05 (0.88, 1.25)0.5858 |
| Q3 | 1.30 (1.11, 1.53)0.0015 | 1.46 (1.23, 1.73)<0.0000 | 1.34 (1.13, 1.59)0.0008 |
| Q4 | 1.82 (1.56, 2.12)<0.0000 | 2.05 (1.75, 2.41)<0.0000 | 1.68 (1.42, 1.99)<0.0000 |
| TyG-WHtR | 1.36 (1.3, 1.43)<0.0000 | 1.35 (1.28, 1.42)<0.0000 | 1.24 (1.17, 1.31)<0.0000 |
| TyG-WHtR quartile |  |  |  |
| Q1 | 1 | 1 | 1 |
| Q2 | 1.06 (0.90, 1.25)0.4972 | 1.12 (0.94, 1.33)0.2005 | 1.12 (0.93, 1.34)0.2212 |
| Q3 | 1.25 (1.06, 1.48)0.0093 | 1.30 (1.09, 1.55)0.0035 | 1.22 (1.02, 1.46)0.0297 |
| Q4 | 2.16 (1.86, 2.51)<0.0000 | 2.15 (1.83, 2.52)<0.0000 | 1.75 (1.47, 2.08)<0.0000 |
| TyG-BMI | 2.84 (2.3, 3.5)<0.0000 | 2.62 (2.12, 3.23)<0.0000 | 2.12 (1.69, 2.64)<0.0000 |
| TyG-BMI quartile |  |  |  |
| Q1 | 1 | 1 | 1 |
| Q2 | 0.84 (0.71, 1)0.046 | 0.87 (0.73, 1.04)0.123 | 0.87 (0.73, 1.04)0.1239 |
| Q3 | 1.17 (1, 1.37)0.0506 | 1.2 (1.02, 1.41)0.0273 | 1.17 (0.99, 1.38)0.0639 |
| Q4 | 1.78 (1.53, 2.07)<0.0000 | 1.73 (1.48, 2.02)<0.0000 | 1.52 (1.29, 1.78)<0.0000 |

Note: OR (95%CI) Pvalue
Non-adjusted model adjust for: None
Adjust I model adjust for: Age; Gender; Race; Survey Year
Adjust II model adjust for: Age; Gender; Race; Family PIR; Survey Year; Education Level; Marital Status; Smoke; Alcohol Drinking; Diabetes
